# The impact of test announcements on subtitle reliance and video comprehension: The role of vocabulary size and working memory

**DOI:** 10.1371/journal.pone.0357500

**Published:** 2026-09-02

**Authors:** Jing Wang, Yuxiao Yang, Fei Chen

**Affiliations:** 1 Foreign Studies College, Hunan Normal University, Changsha, Hunan, China; 2 Shicheng College, Hunan Normal University, Changsha, Hunan, China; 3 Institute of Interdisciplinary Studies, Changsha, Hunan, China; 4 School of Foreign Languages, Hunan University, Changsha, Hunan, China; Bahir Dar University, ETHIOPIA

## Abstract

This study investigates how test announcements affect subtitle reliance and video comprehension among Chinese learners of English, taking into account vocabulary size and working memory variances. Fifty-four participants were recruited in an eye-tracking experiment where they were divided into two groups: a group with a test announcement (TA) of the upcoming comprehension test and a group without (NTA). Results indicated that TA manifested significantly higher total duration of fixation and number of fixations, suggesting heightened subtitle reliance when participants were aware of the test. The average duration of fixation, however, remained similar between groups, implying a comparably minimal cognitive effort in processing L1 texts. For the comprehension test, TA exhibited significantly higher scores for the target video, indicating a positive role of test announcements for video comprehension. Moreover, working memory and vocabulary size were found to be negatively correlated with subtitle reliance only for the TA group, highlighting that these associations may be more evident under test-announcement conditions. These findings were discussed within cognitive theoretical frameworks, furnishing valuable implications for the psychological experiment design and L2 multimedia teaching.

## 1. Introduction

The increasing prevalence of subtitles in video content has garnered considerable attention, as they serve as an effective tool for enhancing viewers’ comprehension, particularly when engaging with content in a second language (L2). Yet, despite extensive research into general subtitle processing and instructional strategies such as test announcements, investigations specifically addressing the impact of test announcements on subtitle reliance and the underlying cognitive mechanism remain sparse. Furthermore, relatively little attention has been paid to individual differences in the existing literature, presenting a notable research gap that this study aims to address.

A test announcement is defined as a formal declaration regarding an impending evaluation or assessment [1]. Prior empirical evidence suggests a correlation between test announcements and heightened attention [2]. Similar correlations have been established between specific pedagogical instructions and cognitive load [3]. While the impact of test announcements has been well-documented in the context of vocabulary acquisition [4] and text reading [5], their effect within the realm of subtitle reliance has yet to be further explored.

Subtitles are increasingly integrated into language learning environments as a mechanism to facilitate Second Language Acquisition (SLA). They play a critical role in enhancing comprehension, vocabulary acquisition, and overall language proficiency [6]. Research indicates that the effectiveness of subtitles often varies according to the learner’s proficiency level, with those exhibiting lower proficiency demonstrating a greater subtitle reliance [7]. It is noteworthy, however, that excessive reliance on subtitles may hinder the development of listening skills, as learners may prioritize reading over listening [8]. As subtitle reliance exerts important influences on SLA, one of the most efficient methods to gauge subtitle reliance is through eye-tracking, a technique employed across various domains of L2 research, which offers insights into actual fixation duration and the real-time comprehension processes during language processing [9]. This technology has also been reported to be helpful in elucidating cognitive mechanisms, including attention, higher-order cognitive processes, and cognitive load during language learning activities [10,11]. Prior research indicated that metrics such as number of fixations (NF), total duration of fixation (TDF), and average duration of fixation (ADF) are informative in revealing attention allocation [12], which could be closely related to subtitle reliance as concerned by the present study.

Research has shown that individual differences can significantly impact L2 acquisition, affecting both the speed and effectiveness of language learning processes [13]. This variability is considered particularly salient in examining how test announcements may affect subtitle reliance across learners. Li et al. [14] elucidated that the interactions between individual differences and overall L2 learning outcomes can be affected by instructional treatments. When it comes to L2 video watching, individual differences such as working memory and vocabulary size may be of great importance, as they are closely related to the management of multimodal cognitive load and the decoding of L2 input [15]. It has been extensively attested that working memory enables simultaneous processing of transient audiovisual stimuli, while larger vocabulary size reduces lexical inferencing demands, freeing resources for integration [13]. Their synergy can also enhance comprehension in dynamic learning contexts [14].

Hitherto, a persistent debate has surrounded the appropriateness of test announcements in both educational and experimental contexts, alongside considerable apprehension regarding the overreliance on subtitles [16]. Furthermore, scant attention has been paid to the specific influences of test announcements on subtitle reliance, with consideration of individual differences. To address this gap, this study employs eye-tracking to examine the impact of test announcements on subtitle reliance and L2 comprehension, taking into account individual differences in working memory and vocabulary size. The findings may offer novel insights into L2 pedagogy as well as the design of future eye-tracking experiments.

### 1.1 Test announcements and subtitle reliance

Subtitles play a pivotal role in facilitating L2 learning by enhancing comprehension, vocabulary acquisition, and exposure to authentic linguistic input. Research demonstrates that subtitled videos provide dual coding of auditory and textual information, allowing learners to cross-reference spoken language with written text, thereby reducing ambiguity and improving retention [17]. For L2 learners, subtitles serve as a scaffold, bridging gaps in auditory processing and enabling access to complex content [7]. However, excessive reliance on subtitles may introduce unintended and alarming drawbacks. It has been assumed that while test announcements may lead to a heightened attention to subtitles and thus improve the immediate task performance, they risk fostering dependency, where learners neglect listening skill development and fail to adapt to real-world L2 interactions without textual support [18].

Since subtitle watching can have a large impact on L2 acquisition, the factors that influence subtitle watching are of great importance. Previous studies have generally confirmed the critical role of pedagogical instructions like test announcements, and the majority of them, which focused on test announcements or other similar instructional practices, have based their research on attention [4,5,19]. Attention can be interpreted as a mechanism for selecting information to be processed in working memory [20]. The correlation between test announcement and attention justifies the validity of the Selective Attention Theory [21]. The theory posits that, as human attention is limited, people tend to selectively attend to certain stimuli that are deemed more important or relevant to their objectives while strategically ignoring other information. Montero Perez et al. [4] demonstrated that learners would intentionally fixate on captions when anticipating vocabulary tests, confirming the role of test announcements as attentional cues. However, studies such as Montero Perez et al. [4] only used L2 subtitles and focused mainly on vocabulary acquisition, without much emphasis on how test announcements affect L2 comprehension. This creates a significant research gap that the present study aims to address.

One of the most commonly used methods to investigate the attention involved in video watching is eye-tracking, as it can tap into uninterrupted, real-time comprehension processes [9]. The effectiveness of eye-tracking measures as indicators of attention is also supported by the “eye-mind link” hypothesis, which posits that eye movements can reflect cognitive and attentional processes [22]. In the context of video watching and text reading, selective attention typically manifests as increased TDF, NF, and ADF, which has been validated by various empirical studies. Kim et al. [2] revealed that announcing a vocabulary test could increase NF on the bottom glosses, reflecting heightened strategic focus. Similarly, Peters et al. [5] observed that explicit instructions (i.e., test announcements) can direct learners’ attention to target words in L2 reading tasks, leading to longer TDF on text regions deemed “test-relevant”. It has also been noted that this selective attention can enhance learning, though in most cases on a surface level, by ensuring that cognitive resources are allocated to meaningful input [23,24]. Nevertheless, the majority of studies on test announcement merely include keyword captions or glosses, necessitating an examination of the effect of test announcement on subtitles.

### 1.2 Test announcements and comprehension

In the context of SLA, the role of test announcements in shaping comprehension outcomes is a topic of significant pedagogical and theoretical interest. The Theory of Planned Behavior provides a useful framework for understanding the potential positive effects of test announcements on comprehension. According to the theory, behavioral intentions are a key determinant of human behavior, and these intentions are influenced by attitudes, subjective norms, and perceived behavioral control [25]. When learners are informed of an upcoming test, they are likely to possess a certain level of conscious awareness [26] and develop a stronger intention to engage with the material strategically, focusing on elements they perceive as relevant to the test, which naturally leads to a better understanding and comprehension of the L2 video content. These tenets were supported by previous literature. Teng and Zhang [27] reported that learners with stronger motivation and self-awareness tend to engage in deeper processing, leading to significantly better performance on language tests. Likewise, Jung and Yang [28] manifested that a test announcement can increase learners’ use of inferencing strategies during video tasks, enhancing overall test performance.

While an increase in comprehension scores may be observed when a test is announced, according to the Theory of Planned Behavior, the announcement of a test can also be detrimental, especially from the perspective of cognitive load. The Cognitive Load Theory [29] elucidates how test announcements may hinder L2 comprehension by amplifying extraneous cognitive load—mental effort expended on non-essential cognitive activities that impede learning [30]. When learners are informed of an impending test, the heightened anxiety and pressure to perform can trigger an extraneous load, exhausting working memory resources and disabling deeper comprehension processes, such as inferencing or contextual integration [31]. Such an effect can even be amplified when the instructions are detailed or of great complexity [32]. The above theories have also been verified by studies, such as Sadeghi and Pourhaji [33], which found that pre-task explicit instructions had the potential to harm overall oral production. To date, few studies have directly examined the effect of test announcements on L2 video comprehension, and a psychological explanation regarding this gap remains overdue.

### 1.3 Individual differences under test announcements

As mentioned earlier, individual differences have been shown to be relevant to L2 acquisition [34]. When viewing L2 videos, vocabulary size and working memory can largely influence learners’ abilities to parse simultaneous audio, text, and visual streams. Learners with larger vocabularies can more readily access word meanings, and those with greater working memory capacity can juggle subtitle decoding alongside visual scene analysis without cognitive overload [35].

It is worth noticing that previous studies like Li et al. [14] have noted that the relationships between individual differences and general L2 outcomes are highly susceptible to different pedagogical instructions, such as test announcements. More importantly, certain correlations tend to be strengthened with explicit instructions compared with implicit instructions. Montero Perez et al. [8] noted that learners with larger vocabulary sizes relied less on subtitles under explicit instructions for basic comprehension. Gass et al. [36] revealed that learners with stronger working memory capacities reduced their reliance on L2 captions after being aware of the upcoming tests. In other words, when explicit instructions are provided, individuals with robust working memory can better integrate auditory input without needing the supplemental visual information offered by subtitles. Taken together, these studies underscore that both extensive vocabulary knowledge and strong working memory lessen the necessity for subtitle support under explicit instruction.

### 1.4 The present study

Given the aforementioned studies, several gaps still remain. First, few studies have examined the impact of test announcements on subtitle reliance and L2 comprehension. Second, the relationships between subtitle reliance and vocabulary size and working memory capacity under different test-announcement conditions have not been thoroughly explored. Addressing these gaps could provide valuable insights into the mechanisms underlying test announcements and their effect on SLA. Based on the above theoretical frameworks and literature review, the following research questions and hypotheses are proposed:

Research Question 1: Do test announcements affect subtitle reliance, as measured by eye-tracking metrics?

Research Question 2: Do test announcements affect L2 comprehension?

Research Question 3: What are the relationships between individual differences (i.e., vocabulary size and working memory) and subtitle reliance under different test-announcement conditions?

Based on the theoretical frameworks and previous findings, for the first research question, it is hypothesized that a test announcement would increase the attention to the subtitles, as demonstrated by an increase in TDF, ADF, and NF. This hypothesis is grounded in Selective Attention Theory and empirical evidence showing that explicit instructions, including test announcements, can direct learners’ attention to task-relevant stimuli [2,5]. For the second research question, the effect of test announcements on L2 comprehension is hypothesized to be bidirectional. On the one hand, informed learners may demonstrate improved comprehension due to heightened strategic attention to subtitles, as posited by the Theory of Planned Behavior. On the other hand, test announcements could impair comprehension by inducing extraneous cognitive load, as proposed by Cognitive Load Theory. Regarding the third research question, vocabulary size and working memory were expected to be negatively related to subtitle reliance, with these negative relationships hypothesized to be more evident when a test was announced, as suggested by previous studies [14].

## 2. Methods

### 2.1 Participants

An a priori power analysis was conducted using G*Power 3.1.9.7 [37] for a two-group within-between interaction ANOVA, with the alpha level set at  .05, the desired statistical power at  .80, and the effect size specified as medium (f = 0.25) [38]. The analysis indicated that a minimum total sample of 34 participants was required, corresponding to 17 participants in each group. To enhance the robustness of the group comparisons and to allow for potential data loss, a substantially larger sample of 54 Chinese EFL learners (48 females and 6 males) were recruited, aged from 17.00 to 21.25 (Mage = 19.31, SD = 0.94). All participants were undergraduate English majors recruited from a normal university in China. They were divided into a Test Announcement (TA) group (n = 28) and a Non-Test Announcement (NTA) group (n = 26) based on comparable between-group differences of English proficiency (measured by Quick Placement Test), vocabulary size (LAIX [39]) and working memory capacity (Digit Span Test). Ethical approval for this study was granted by the Biomedical Research Ethics Committee of Hunan Normal University (Ethics Approval No. 609 [2025]). Written informed consent was obtained from all participants before the study began. All participants reported no history of speech or hearing disorders. Participation was voluntary, confidentiality was assured, and monetary compensation was provided. The differences in age, English learning experience, vocabulary size, working memory, and English proficiency between the two groups were statistically computed through Linear Mixed Effects Models (LMMs). Results showed no significant difference between the two groups in any of these metrics, as demonstrated in Table 1.

**Table 1:** Demographic characteristics of the TA and NTA groups. WM scores = scores of the working memory test, QPT Scores = scores of the Quick Placement Test (English proficiency).

Demographic Characteristics	TA	NTA	p
Number	28	26	
Age (SD)	19.42 (1.02)	19.20 (0.84)	0.22
Learning Experience (SD)	10.71 (2.28)	11.05 (1.89)	0.58
Vocabulary Size (SD)	5630.82 (1555.58)	5846.71 (1315.45)	0.24
Working Memory (SD)	24.04 (3.24)	23.04 (3.28)	0.13
QPT (SD)	30.40 (3.68)	30.16 (6.19)	0.33

### 2.2 Audio-visual materials

A control video was included to ensure that the baseline subtitle reliance is consistent across groups, thereby verifying that any observed differences in subtitle reliance in the target videos resulted from the specific announcement conditions rather than pre-existing group differences. The control video (duration: 3.11 minutes) was an excerpt from a YouTube video titled “2 Hours of English Conversation Practice - Improve Speaking Skills”, posted by Learn English with English Class 101. In this video, two native Americans discuss things they miss about America. The target video was derived from an adaptation of a passage from the TOEFL speaking section’s Task 4, namely the “Experimenter Effect” (duration: 3.10 minutes). It was recorded in the form of an interview by a Canadian linguist and a Chinese phonetician in a sound-attenuated classroom. The recording setup included a microphone with an external sound card and a wall-mounted camera positioned approximately four meters from the speaker. Both the control and target videos featured Chinese subtitles in Huakang Jingang boldface, sized at 22 points. The subtitles were white and presented on a single line, horizontally centered in a fixed position within the black band at the bottom of the video. No outline, shadow, or other special text effects were applied. Subtitle onset and offset were synchronized with the corresponding spoken segments, and presentation duration therefore varied according to the duration of the speech represented by each subtitle. The same subtitle formatting and positioning were used for both videos. The subtitle design followed the BBC Subtitle Guidelines [40]. The two videos were designed to be similar in length and difficulty level. Linguistic analysis by RANGE software [41] confirmed that 94.24% of the vocabulary was from the most frequent 2,000-word families, and 2.25% was from the 3,000-word level.

Each video was followed by a five-item, four-option multiple-choice comprehension test. The items were designed to assess learners’ understanding of central ideas, explicitly stated information, and inferential or integrative content. Each correct answer received one point, yielding a maximum score of five. Together, the items provided a concise and structured content-based measure of video comprehension. The transcripts of the two videos and comprehension tests are available in S2 Appendix.

### 2.3 Procedure

**Fig 1:**
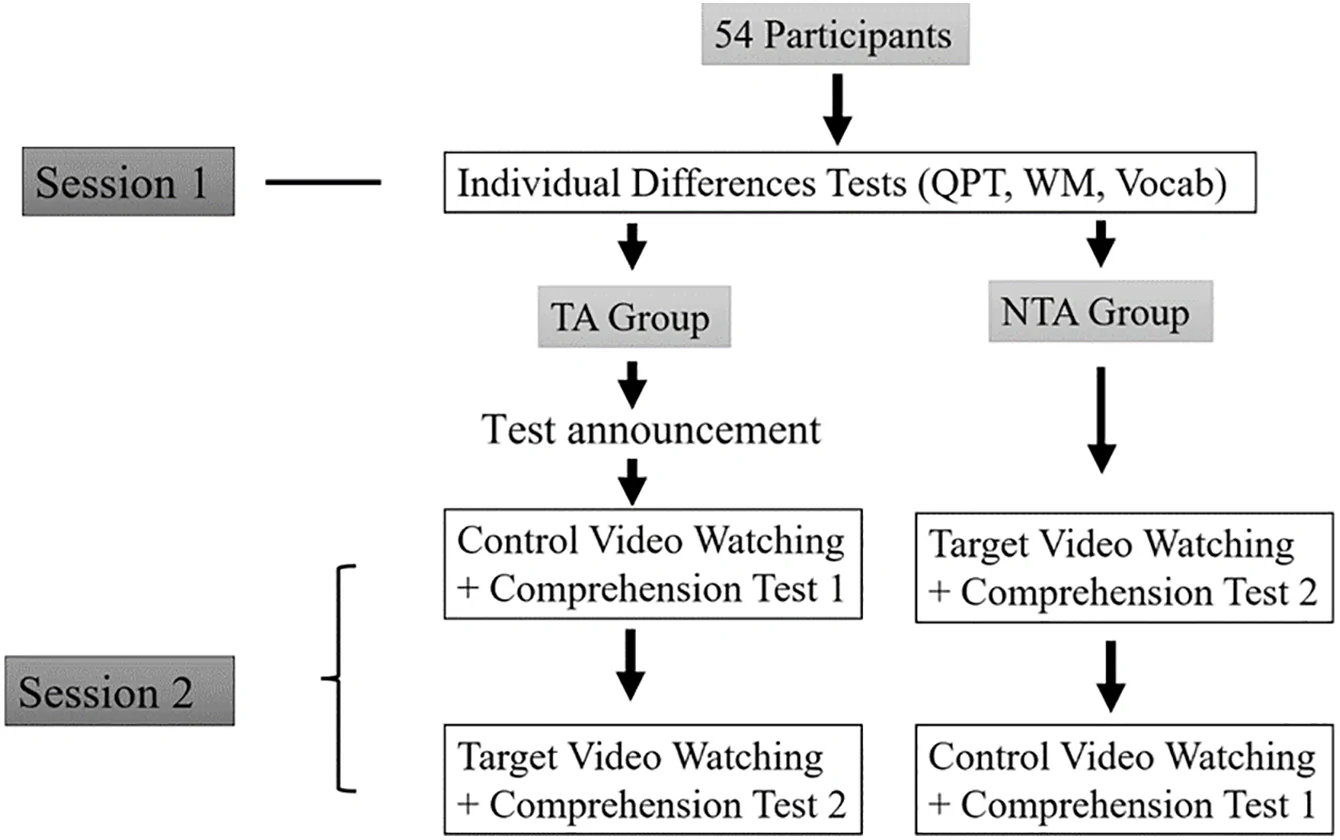
Visual diagram of the research procedure. QPT = Quick Placement Test, WM = working memory, Vocab = vocabulary size, TA = test announcement, NTA = non-test announcement.

The data collection process was divided into two sessions. In session 1, participants were asked to work on the individual difference tests. In session 2, participants were required to watch both control and target videos, and finish the comprehension tests respectively. For the TA group, the participants were informed that there would be a comprehension test after watching the video. They first finished the control video, and then the target video. In contrast, the NTA group was not told about the comprehension test, and they first finished the target video, and then the control video. The target video was played first for the NTA group because if the participants finished the control video first, they would be expecting a comprehension test for the target video as well, which might alter their watching behaviors. The experiment procedure is delineated in Fig 1.

For the collection of individual difference data, the participants first took a vocabulary test (LAIX test) and then the Quick Placement Test (QPT) [42]. The vocabulary test lasted 3 minutes, while the QPT took approximately 30 minutes. The QPT test comprises 60 multiple-choice questions that evaluate overall language proficiency. Each question is worth one point. Following the instruction of the test, the scoring system is tiered: if a student scores less than 36 points on the first 40 questions, the last 20 questions will not be considered, and their final score will be based only on the first 40 questions. The results can be reported as a CEFR (the Common European Framework of Reference) level [43]: scores of 0–10 align with A1, 11–20 with A2, 21–30 with B1, 31–40 with B2, 41–50 with C1, and 51–60 with C2. The working memory test (Digit Span Task) was administered in a separate room to minimize distractions, with stimuli presented through headphones. Participants would repeat the digits aloud, first in the original order (Forward Digit Span) and then in reverse (Backward Digit Span); the responses were scored by an experimenter. One point is given for each fully correct sequence (no omissions or errors) out of a possible 32 points. Responses were recorded using Cool Edit Pro and saved as WAV files for later verification, with the entire task lasting 15–20 minutes. Before the video-watching session, participants were assigned to either the TA group (n = 28) or the NTA group (n = 26) in a manner that balanced the between-group individual differences.

In session 2, the eye-tracking experiments took place in a quiet, well-lit language lab, with the curtains closed and with one participant and one experimenter present at a time. For the TA group, participants were explicitly informed about an upcoming reading comprehension test, with test papers visibly placed on the desk. In contrast, the NTA group received no such information, with test materials being placed out of sight. To ensure the confidentiality of the test, participants in the NTA group signed a non-disclosure agreement and were assigned to different rooms before and after the experiments, so that those who had completed the tests would not encounter those who had not. Additionally, participants’ mobile phones were collected while waiting to avoid potential communication about the tests. During the experiments, participants were seated comfortably and instructed to minimize head movements to ensure accurate eye-tracking. A Tobii Spectrum desktop eye-tracker (with a 1200 Hz sampling rate and binocular recording) was used, displaying videos on a 23.8-inch monitor (1920 × 1080 resolution). Right before playing the video, participants were requested to undergo a nine-point calibration to ensure the validity of the recorded eye movement data. The Tobii Eyetrackers automatically recorded fixation data and pupil diameter (binocular average in millimeters). Both groups viewed the same videos under the same laboratory lighting conditions. After watching the videos, both groups completed the corresponding comprehension tests, which were later scored by the experimenter.

### 2.4 Data analyses

The eye-tracking data, including TDF, NF, and ADF within the AOIs, were analyzed using Tobii Pro Lab (Version 1.232, 2023). The data were managed, visualized, and analyzed using R 4.3.3 [44]. In this study, continuous dependent variables were analyzed with Linear Mixed Models (LMMs), whereas binary dependent variables coded as 1/0 were analyzed with Generalized Linear Mixed Models (GLMMs). LMMs and GLMMs were fitted using the “lme4” package (version 1.1-35.1) [45], with p-values obtained via the “lmerTest” package (version 3.1.3) [46]. By “Participant” and “Item” random slopes were prioritized, unless model convergence issues arose or likelihood ratio tests indicated no significant improvement over models with random intercepts [47]. Main effects and interactions were assessed with Type II Wald chi-square tests using the “car” package (version 3.1−2) [48]. The pairwise comparisons were performed with the “emmeans” package (version 1.10.0) [49]. For the analysis of the relationships between subtitle reliance and individual difference variables, Pearson correlations [50] were performed. Average pupil diameter was examined as a supplementary measure using an LMM between-group comparison.

## 3. Results

### 3.1 Effect of test announcements on subtitle reliance

**Fig 2:**
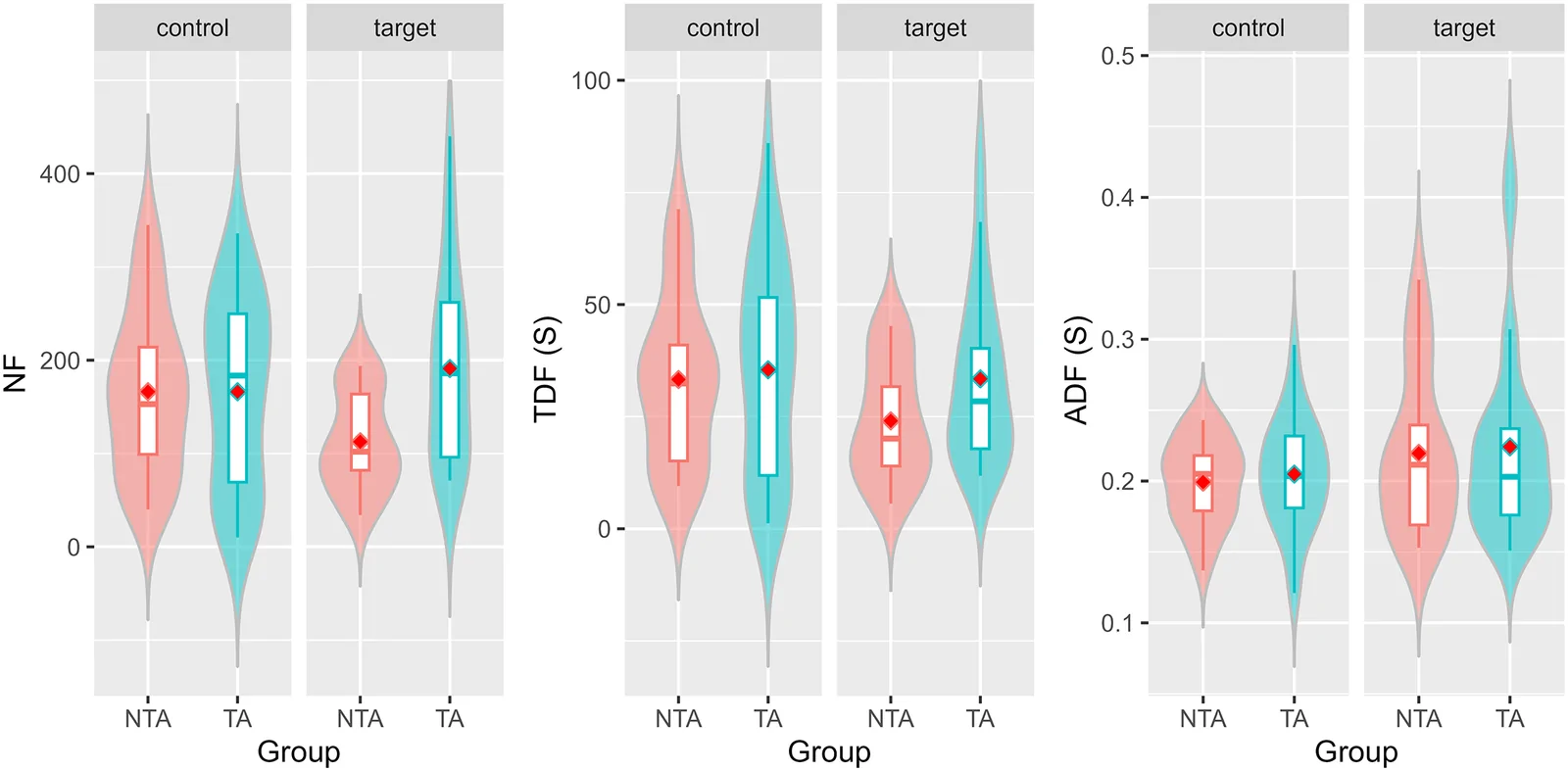
Box-Violin Plot of the NF, TDF, and ADF between the two groups (NTA and TA) and the two videos (control and target). NF = Number of Fixation, TDF = Total Duration of Fixation, ADF = Average Duration of Fixation, TA = test announcement group, NTA = non-test announcement group. The diamond shape represents the mean.

To investigate the impact of test announcements on subtitle reliance, the eye-tracking metrics of NF, TDF, and ADF were analyzed across the two groups for both control and target videos. Results are demonstrated in Fig 2.

For the control video, all three metrics remained consistent between the two groups, indicating comparable baseline subtitle reliance. In contrast, for the target video, the NTA group showed markedly fewer NF and TDF compared with the TA group. That said, the NTA group and the TA group exhibit relatively similar ADF values (Table 2).

**Table 2:** Means and standard deviations (SD) of subtitle reliance as indexed by eye-tracking metrics. VT = Video Type, NF = Number of Fixations, TDF = Total Duration of Fixation, ADF = Average Duration of Fixation, TA = test announcement group, NTA = non-test Announcement group.

VT	Metric	NTA		TA	
		Mean	SD	Mean	SD
control	NF	166.24	87.47	166.2	101.15
	TDF	33.29	18.4	35.46	23.35
	ADF	0.20	0.03	0.20	0.04
target	NF	112.59	52.35	191.08	102.82
	TDF	29.04	26.67	46.47	35.32
	ADF	0.22	0.06	0.22	0.07

For inferential statistics, LMMs were fitted for each eye-tracking metric. Regarding NF, an LMM was fitted with “NF” as the dependent variable, “Group” and “Video Type” as the fixed effects, “Participant” as the random intercept, and “Vocabulary Size” and “Working Memory” as covariates. The model revealed a significant interaction between “Group” and “Video Type” (χ² (1) = 8.31, p < .05). Post-hoc comparisons (see Table 3) manifested that while no significant difference was found in the control video (p = .92, d = −0.04), the NTA group had significantly fewer fixations than the TA group in the target video (p < .05, d = −1.21).

**Table 3:** Post-hoc pairwise comparison of TDF and NF as functions of Group and Video Type. VT = Video Type, NF = Number of Fixations, TDF = Total Duration of Fixation.

Metric	VT	Group contrast	estimate	SE	df	t.ratio	p.value	cohens'd	95% CI
NF	control	NTA – TA	-2.51	24.30	88.50	-0.10	.92	-0.04	[-0.91, 0.82]
	target	NTA – TA	-67.80	26.10	100.90	-2.60	.01^*^	-1.21	[-2.15, -0.27]
TDF	control	NTA – TA	-3.08	6.96	102.20	-0.44	.66	-0.15	[-0.83, 0.53]
	target	NTA – TA	-16.54	7.68	109.80	-2.15	.03^*^	-0.82	[-1.58, -0.06]

Signif. codes: 0 ‘***’ 0.001 ‘**’ 0.01 ‘*’ 0.05 ‘.’ 0.1 ‘ ’ 1

For TDF, an LMM was fitted with the same fixed, random effects, and covariates as the model for NF, but with “TDF” as the dependent variable. The LMM indicated a marginal interaction between the two fixed effects (χ² (1) = 2.75, p = .097). Pairwise comparisons (Table 3) revealed that while no significant difference was observed in the control video (p = .66, d = −0.15), in the target video, the NTA group had significantly shorter TDF than the TA group (p < .05, d = −0.82).

For ADF, an LMM was fitted with “ADF” as the dependent variable, and the same fixed, random effects, and covariates as the previous two models. The LMM revealed no significant main effect of “Group” (χ² (1) = 0.20, p = .65) nor a significant interaction between “Group” and “Video Type” (χ² (1) = 0.04, p = .85). This indicated that the test announcement had little impact on ADF, as no significant difference was found between TA and NTA (β = 0.006 ± 0.01 SE, t = 0.49, p = .63).

In summary, the results revealed that the two groups had comparable baselines in subtitle reliance, as no differences were detected across the three eye-tracking metrics in the control video. For the target video, on the other hand, the test announcement significantly increased subtitle reliance, as manifested by the TDF and NF. That being said, such an increase was not found in ADF.

### 3.2 Comprehension scores under different test announcement conditions

**Fig 3:**
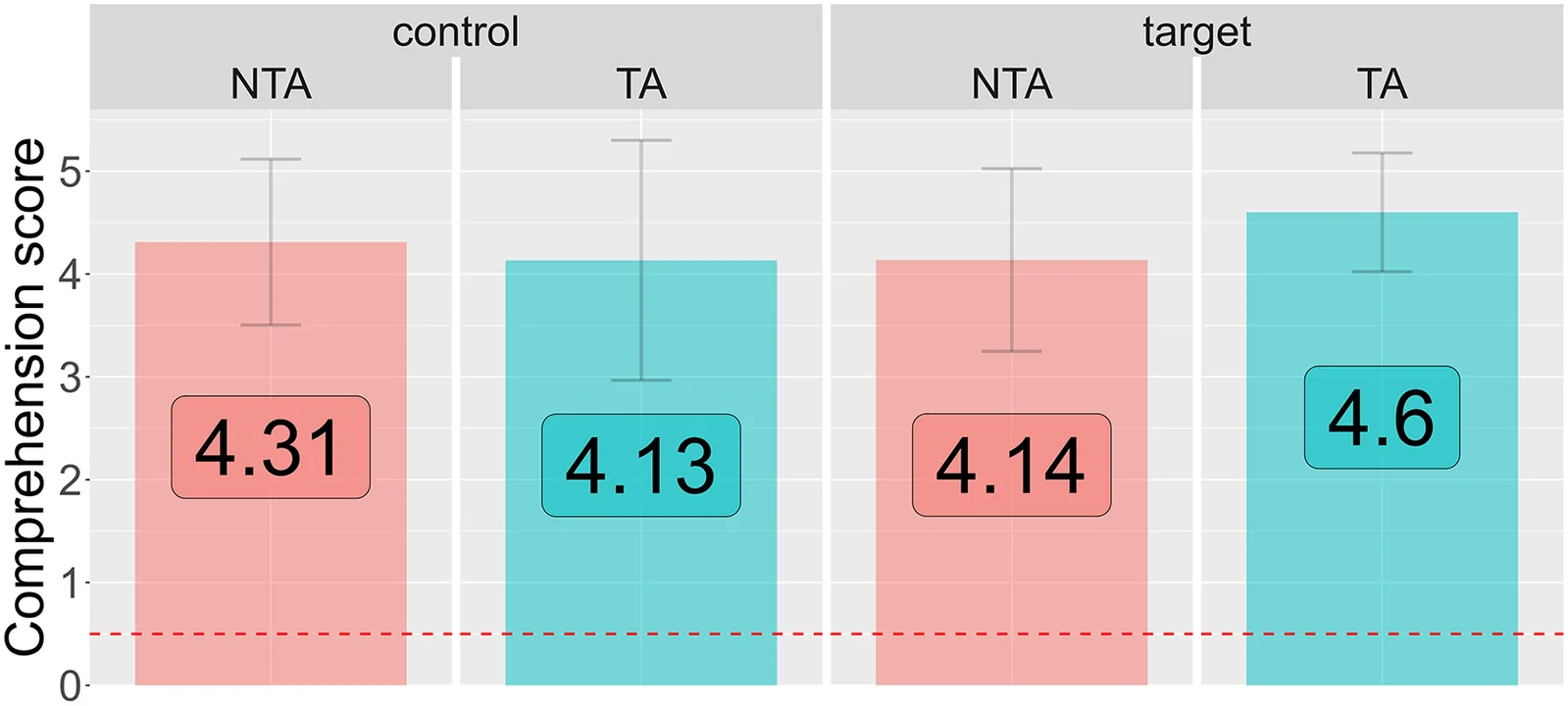
The bar plot of the comprehension test score. TA = test announcement group, NTA = non-test announcement group.

To investigate whether the announcement of a test would impact video comprehension, the scores of the two groups were statistically compared. For the control video, the performance was similar between the two groups; the NTA group scored an average of 4.31 out of 5 (SD = 0.81), while the TA group scored 4.13 out of 5 (SD = 1.17). For the target video, the TA group achieved visibly higher accuracy (mean = 4.60, SD = 0.58) compared to the NTA group (mean = 4.14, SD = 0.89) (Fig 3).

For deductive statistics, a GLMM was fitted for the comprehension test scores. With “Group” and “Video Type” as the fixed effects, and “Participant” and “Item” as the random intercepts; “Score” was calculated as the binomial dependent variable (correct = 1, incorrect = 0). The GLMM revealed a significant interaction between “Group” and “Video Type” (χ² (1) = 5.02, p < .05). The post-hoc comparison further demonstrated that whilst no significant between-group difference was observed in the control video (β = 0.07 ± 0.40 SE, z = 0.17, p = .87, OR = 1.07, 95% CI [0.49, 2.35]), the NTA group had scored significantly lower than the TA group in the target video (β = −1.15 ± 0.49 SE, z = −2.34, p < .05, OR = 0.32, 95% CI [0.12, 0.83]). The results indicated that the presence of a test announcement had significantly enhanced the L2 comprehension.

### 3.3 Correlation between subtitle reliance and individual differences

**Fig 4:**
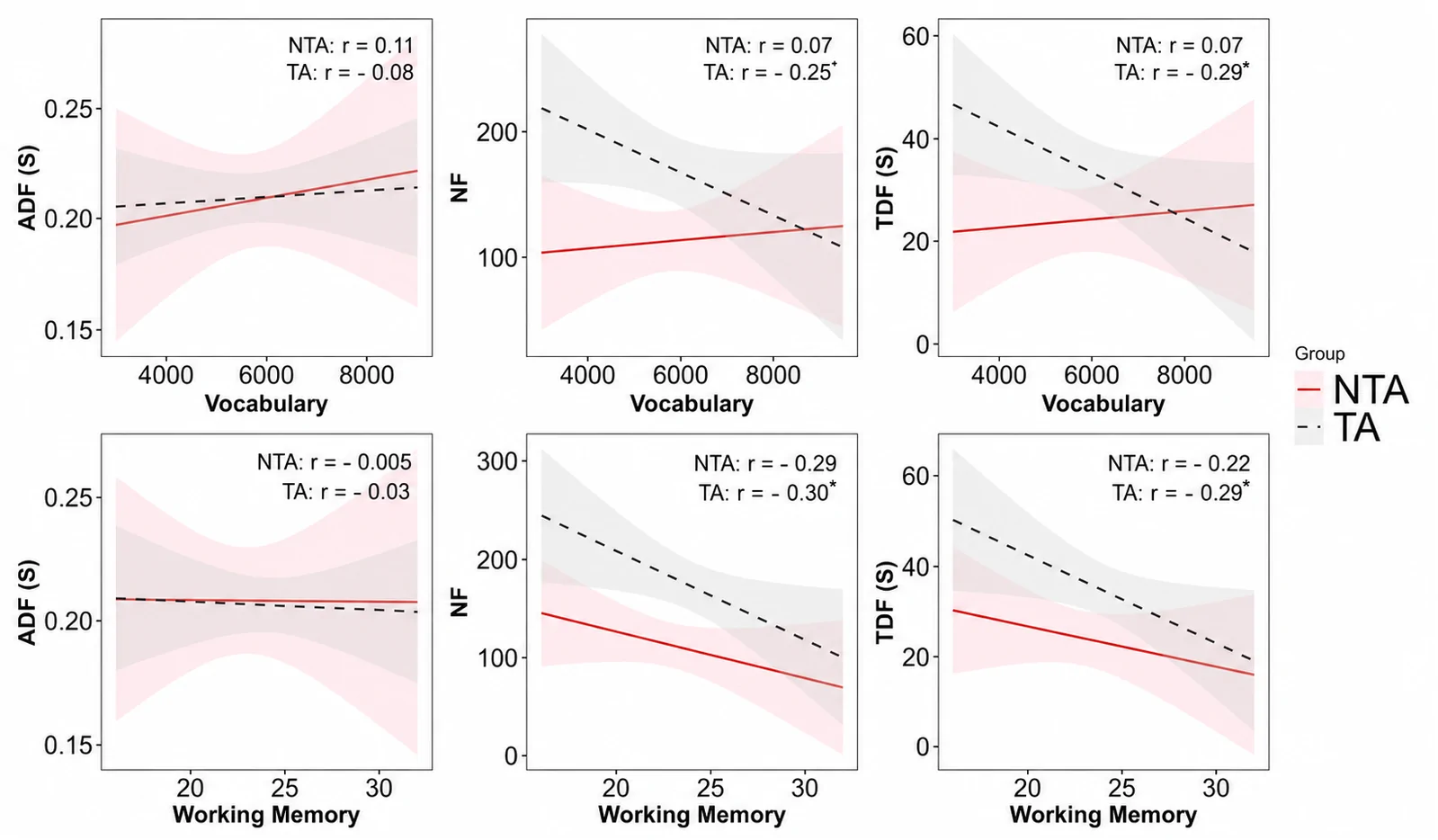
Correlation plots between eye-tracking metrics and vocabulary size/working memory. ADF = average duration of fixation, NF = number of fixations, TDF = total duration of fixation, TA = test announcement group, NTA = non-test announcement group.

To address Research Question 3, Pearson correlations were calculated separately for the TA and NTA groups to examine the relationships of vocabulary size and working memory with the three eye-tracking metrics of subtitle reliance. As depicted in Fig 4, for the NTA group, no significant correlations were found between vocabulary size and any of the eye-tracking metrics (ps > .05), nor between working memory and any of the eye-tracking metrics (ps > .05). However, in the TA group, negative correlations were found between NF and vocabulary size (r = −0.25, p < .1) and between NF and working memory (r = −0.30, p < .05). Similarly, negative correlations were found between TDF and vocabulary size (r = −0.29, p < .05) and between TDF and working memory (r = −0.29, p < .05). That is to say, when tests were announced, participants with larger vocabulary sizes and working memory capacities would generally focus less on the subtitles. Notably, no correlation was identified between ADF and both vocabulary size and working memory in either group.

These results show that negative associations of vocabulary size and working memory with NF and TDF were observed in the TA group but not in the NTA group, suggesting that these associations may be more evident under test-announcement conditions. By contrast, no significant associations of ADF with either vocabulary size or working memory were found in either group.

## 4. Discussion

The purpose of this study was to examine the effect of test announcements on subtitle reliance and L2 video comprehension among Chinese EFL learners with consideration of individual differences in the process. Results showed that the TA group exhibited significantly higher TDF and NF on subtitles compared with the NTA group, which mostly supported our first hypothesis that test announcements would increase attention to subtitles. In terms of L2 comprehension scores, the TA group achieved significantly higher scores, supporting our second hypothesis and yielding a pattern consistent with the Theory of Planned Behavior and Selective Attention Theory. Regarding individual differences, vocabulary size and working memory showed significant negative correlations with TDF and NF only in the TA group, indicating a condition-dependent pattern in the associations between individual differences and subtitle reliance. The following sections provide a detailed discussion of these findings and explore their theoretical and practical implications.

### 4.1 Test announcements raise subtitle reliance

The present study demonstrated that test announcements significantly increased subtitle reliance among L2 learners, as evidenced by heightened TDF and NF in the TA group compared to the NTA group. These findings are consistent with Selective Attention Theory, which posits that explicit instructions, such as test announcements, direct learners’ attention toward task-relevant stimuli—in this case, subtitles. The increased TDF and NF suggest that the participants strategically allocated more cognitive resources to subtitles when anticipating a comprehension test, perceiving them as critical for task success. This strategic behavior mirrors previous observations by Kim et al. [2], who reported that announcing a vocabulary test increased fixation counts on glossed text, reflecting learners’ deliberate focus on perceived “test-relevant” elements.

The findings of this study underscore the critical role of experimental instructions in shaping participants’ behavioral patterns. Specifically, the observed increase in subtitle reliance under the TA condition aligns with broader evidence suggesting that explicit instructions or contextual cues (e.g., test announcements) act as demand characteristics [51], guiding participants toward behaviors expected by the experimenter. Rosenthal [52] also calls for attention to the fact that subtle procedural cues, such as informing participants about an upcoming test, can significantly alter their cognitive strategies, leading to artificial or strategic responses rather than naturalistic engagement. Even though explicit instructions like test announcements may improve test performances, researchers who aim to capture authentic or naturalistic behavioral data in multimedia learning contexts should minimize explicit test-related instructions to avoid inducing task-specific attentional biases if their purpose is to observe naturalistic behaviors.

Regarding the lack of significant differences in ADF between the two groups, three factors may contribute to this result. First, the subtitles in this study were presented in participants’ L1, which requires shorter processing time compared to L2 texts. In the present study, the ADF values (approximately 0.2 seconds) align with the established norms for L1 reading, where fixation durations average 200–250 milliseconds [53]. This contrasts with L2 processing, where fixations often exceed 300 milliseconds due to the additional cognitive effort required for decoding unfamiliar orthography or syntax [54]. That is to say, due to the relative simplicity of L1 subtitles, participants can attain understanding most easily, leaving little room for ADF to fluctuate.

Additionally, the low linguistic and conceptual complexity of the video content (94.24% of the vocabulary from the most frequent 2,000-word families) likely reduced the need for prolonged ADF. Simplistic input allows learners to extract information rapidly, diminishing the necessity for extended processing at individual fixation points.

Moreover, the dynamic nature of video stimuli can be the underlying reason. Different from static text reading discussed in previous studies, where learners were able to control pacing, re-read, and dwell longer on complex structures [2], videos, in contrast, impose temporal constraints; participants cannot freely control the pacing of information, as the subtitles are aligned with the visual-audio content, which automatically proceeds to the next line with time. Prolonged fixations risk missing subsequent auditory or visual cues, compelling learners to prioritize frequent, brief fixations over deeper processing at specific points [55]. Thus, the observed increase in TDF for the TA group primarily stemmed from elevated NF rather than ADF, as participants compensated for time-limited fixations by distributing attention to a larger number of fixations.

### 4.2 Test announcements enhance L2 comprehension

The findings of this study highlight that test announcements can significantly enhance L2 comprehension, which is consistent with the Theory of Planned Behavior. According to the theory, learners’ behavioral intentions—shaped by their perceived relevance of a task, motivation, and strategic goal-setting—drive engagement with instructional materials. When informed of an upcoming comprehension test (TA condition), participants in this study may have developed stronger intentions to actively process subtitled content, focusing on extracting meaning and contextual information rather than merely memorizing surface-level elements, such as isolated vocabulary forms, which aligns with Schmidt’s [56] claim regarding the facilitative role of test announcements. Unlike prior studies that emphasized the impact of test announcements on surface-level processing, such as word form recognition [4,5], the current results demonstrate that test announcements can also improve overall comprehension, as evidenced by higher comprehension scores on the content-based test.

Contrary to the predictions derived from the Cognitive Load Theory, which posits that participants’ comprehension performances might be dampened by the possible extraneous cognitive load (mental effort expended on non-essential cognitive activities) caused by the test announcement [57], the present study found no evidence for this hypothesis. First, the comprehension score of the target video was significantly higher for the TA group, indicating that the test announcement was facilitative, rather than imposing an overwhelming cognitive load that hampers performance.

Second, evidence can be found through the participants’ average pupil diameter, a well-established physiological indicator of cognitive load; the higher the value, the greater the cognitive load [58]. In this study, the average pupil diameter appeared to be similar between the TA group (mean = 3.95, SD = 0.50) and the NTA group (mean = 4.13, SD = 0.53) for the target video, and statistical comparisons of an LMM manifested no significant difference between the two groups (β = 0.12 ± 0.12 SE, t = 1.07, p = .29, d = 0.71, 95% CI [−0.62, 2.04]) in the target video. The non-significant result indicates that the present study did not detect a reliable between-group difference in average pupil diameter. Thus, the higher comprehension score in the TA group was not accompanied by a statistically detectable increase in this physiological index of cognitive load. Taken together, these findings suggest that the test announcement did not impose a statistically detectable additional cognitive burden as indexed by average pupil diameter. Nevertheless, this non-significant result should be interpreted cautiously and should not be taken as evidence that cognitive load was equally the same between the two groups.

One possible explanation for the non-extraneous cognitive load may lie in the simplicity of the announcement. The fact that participants were only informed of an upcoming comprehension test without any further details (e.g., specific focus areas or performance benchmarks) reduced extraneous demands on working memory. This contrasts with studies employing complex task instructions, such as Robinson [31], where detailed instructions (e.g., “focus on verb tenses”) fragmented attention, thereby exhausting cognitive resources. Similarly, Schroeder [3] reports that announcements requiring learners to monitor multiple linguistic features (e.g., grammar and prosody) increased extraneous load, impairing comprehension. In the current study, the minimalist announcement (“there will be a comprehension test”) provided a clear yet flexible goal, allowing learners to autonomously balance attention between subtitles and auditory input without rigid constraints. As a result, the TA group achieved higher comprehension scores without a stark increase in cognitive load.

Pedagogically, these results suggest simple, goal-oriented test announcements in L2 instruction. By signaling task relevance without imposing excessive demands, educators may support learners’ strategic engagement while mitigating the risk of heavy cognitive load. In sum, test announcements, when designed thoughtfully, may represent a low-cost tool for enhancing L2 comprehension without necessarily compromising cognitive efficiency.

### 4.3 Condition-dependent relationships between individual differences and subtitle reliance

The role of individual differences appeared to be a critical factor in this study. Vocabulary size exhibited a significant negative correlation with TDF and NF in the TA group, consistent with Montero Perez et al. [8], who argued that learners with larger vocabularies rely less on captions when explicit instructions, such as test announcements, are given. This suggests that learners with stronger lexical knowledge are better equipped to process auditory input independently, reducing their need for subtitle reading. In the NTA group, however, no significant correlation between vocabulary size and subtitle reliance was observed. This condition-dependent pattern also aligns with Li et al. [14], who found that individual differences exert stronger effects under explicit instructional conditions, as learners’ cognitive resources are more deliberately allocated.

The study also revealed a significant negative correlation between working memory and NF and TDF in the TA group, supporting Gass et al. [36], who found that learners with higher working memory capacity, when provided with explicit instructional cues, depended significantly less on subtitles. This further confirms that working memory tends to facilitate the integration of auditory and visual input, reducing the need for textual support. Like vocabulary size, the relationship between working memory and subtitle reliance was observed only in the TA group, suggesting that explicit instructions may make the role of individual differences in learning strategies more evident [14].

One possible explanation for why these individual differences emerged only under the TA condition concerns the cognitive and attentional mechanisms associated with explicit instructional cues. This condition-dependent pattern may be understood through the lens of attention regulation and strategic allocation of cognitive resources. When learners are informed of an impending test, they may shift from a more passive, incidental processing mode to an evaluative, goal-oriented mindset, engaging top-down attentional control mechanisms [59]. In contrast, without explicit cues, learners may default to passive, bottom-up processing, where individual differences play a diminished role as attention is diffused across multimodal inputs [60].

Notably, ADF showed no correlation with any individual difference variables in either group, reinforcing its sensitivity to task complexity rather than individual traits. As Rayner et al. [61] noted, ADF reflects processing difficulty, and the stimuli in this study may not have sufficiently challenged participants to elicit variance. Thus, future research may turn to more challenging audiovisual materials or L2 subtitles to further examine ADF.

### 4.4 Pedagogical and empirical implications of test announcements

The findings of this study suggest that a simple test announcement—merely informing learners of an upcoming comprehension task— can direct learners’ attention to target content, enhancing L2 comprehension outcomes. Educators may therefore consider using relatively concise advance information to guide L2 learners through video watching.

While test announcements improved comprehension in this study, the gains were achieved through heightened subtitle reliance, which still carries pedagogical trade-offs. Excessive dependence on subtitles may hinder the development of auditory processing and inferencing skills critical for real-world L2 interaction. As Danan [16] warns, prolonged subtitle use fosters a “crutch effect,” where learners prioritize reading over listening, potentially delaying the automation of phonological decoding (procedural knowledge).

This study highlights how instructional cues, such as test announcements, can significantly impact test performance and experimental validity. The risk of unintended priming might blur the line between authentic learning processes and artificially induced strategies, potentially undermining the ecological validity of the results. Thus, researchers must carefully assess whether observed effects represent natural cognitive mechanisms or responses specific to the experiment.

## 5. Conclusion

This study examined the effect of test announcements on Chinese EFL learners’ reliance on subtitles and their L2 video comprehension, taking into account individual differences of working memory and vocabulary size. The research mainly aimed to determine whether alerting learners to an impending test would alter their subtitle reliance and L2 comprehension outcomes during video-based tasks. It also examined the condition-dependent associations between subtitle reliance and individual-difference variables.

The findings indicate that participants in the test announcement group exhibited significantly higher NF and longer TDF on subtitles. This enhanced subtitle engagement was accompanied by higher comprehension scores, suggesting that test announcements serve as an effective cognitive trigger. Furthermore, learners with larger vocabularies and stronger working memory capacities tended to rely less on subtitles in the TA group, whereas no significant associations were observed in the NTA group. This condition-dependent pattern suggests that these associations may have been more evident when participants were informed of the upcoming test.

These results furnish important implications for L2 instructional design. Test announcements can be strategically used to guide learners’ attention toward critical linguistic information in multimedia environments, thus enhancing comprehension. Therefore, educators may benefit greatly from incorporating well-timed and simple test cues into their teaching materials. Yet, it is also noteworthy that such practice has the potential to distort the experiment outcomes.

Several limitations should be considered. The sample was relatively modest and consisted exclusively of Chinese EFL learners majoring in English at one university, which may limit the generalizability of the findings. The study did not directly measure motivation, anxiety, test expectancy, behavioral intention, or attentional regulation, so the psychological processes proposed in the Discussion remain interpretive. Also, the average pupil diameter was used as an indirect supplementary measure, and the non-significant between-group difference should not be interpreted as evidence of equivalent cognitive load, which may require further exploration. Finally, because the study did not formally test interaction effects, the condition-specific correlations should not be interpreted as definitive evidence that test announcements statistically moderated these associations.

For future directions, this research employed only one type of test and focused solely on working memory and vocabulary as indices of individual differences. Future studies can include more tests, be they listening tests or vocabulary tests. Meanwhile, more individual differences, like executive functions, can be explored. Moreover, varying the complexity of test announcements and examining different subtitle configurations (e.g., L1, L2, bilingual) could also elucidate the effects of test announcements on L2 video watching. Future work should also recruit broader samples, directly measure motivation, anxiety, test expectancy, and other affective variables, and formally test interactions between test-announcement condition and individual differences.

In conclusion, the study advances the understanding of the impact of test announcements on subtitle reliance and L2 comprehension and highlights the condition-dependent associations of vocabulary size and working memory with subtitle reliance. While offering promising pedagogical strategies, the research also opens avenues for future investigation to refine the application of test announcements.

## Supporting information

S1 Dataset Anonymized dataset. (CSV)

S2 Appendix Supplementary materials containing the visual-stimulus scripts and comprehension tests. (DOCX)
