# Supplementary material for: The impact of test announcements on subtitle reliance and video comprehension: The role of vocabulary size and working memory
Source: PLoS One. 2026 Sep 2;21(9):e0357500. doi: 10.1371/journal.pone.0357500 (PMC13537685; doi:10.1371/journal.pone.0357500)
Supplement: Supplementary file 1 [file pone.0357500.s002.docx]

Supplementary Materials

# APPENDIX A

**Script A.1 (The script of the visual stimuli)**

**Things We Miss about America (Control Video)**

A: Hi, everybody! Welcome back. My name is Alicia and I'm joined today in the studio by Michael.

B: Hey, everybody!

A: And today our topic is going to be our things, our favorite things that we miss when we are outside of America. So, let’s get right into it. The first thing on my list for today, my list of three items, I’ve chosen ovens. One of the things that I really miss when I’m outside of America is ovens. The ovens that my family has always had in our house are very, very big. You can cook turkeys. You can cook casseroles. You can cook pretty much anything you could ever imagine in an oven. When I’m outside of America I often find that oven sizes are noticeably smaller or ovens themselves are just harder to come by than they are in America. So that’s one thing that I really, really miss when I’m outside the States. I don’t know if this is a thing that plays a big part in your life, but for me it’s a big thing.

B: Hmm...honestly, I didn’t notice until you said that because I don’t cook. I’m lazy I just go to the convenience store I go to, like whatever, street vendor or something like that, which I know is more expensive. But you know, as a true American I just want convenience, you know. Yeah, but I think most things like you said, I think that’s gonna be a general theme for most of us, is everything’s bigger in America. So, well, actually that leads me to one. It’s a good transition. Let me pick the one, this one, okay, so speaking of everything being bigger. Space. I miss space. You take it for granted. You don’t realize it especially if you go to some more crowded countries, like in Asia, and that’s where I’ve been for the past while, yeah, most people you have no idea. Um in America it’s pretty common. I would say it’s starting to change but it’s typical for 18-year-olds to move out and live by themselves. And that’s not true in a lot of countries and in a lot of countries, they live with their parents until they get married. And so, I grew up going to house parties. You don’t go to the bar. You can’t drink legally until you’re 21. So, you go to people’s houses because it’s cheaper even when you are of age as you should wait. But you know, there’s always house parties. There’s never an issue to have space, also driving. There are tons of space, tons of room. Yeah, just in general, I miss space. So that’s probably why they don’t have ovens and on some other countries.

A: Right, right, yeah. Your point about space is so true, especially that point that you made about the difference between having house parties and parties at a bar, at a restaurant. When I’ve been in countries in Asia as well, nope, I rarely get invited to people’s houses. You only get invited to someone’s house if you’ve known them for a long time and your pretty close friends, at least in my case. Maybe my friends are different from your friends. I don’t know. But yeah, having a house party is very, very rare I find. And if you do have a house party, there are very few people. It’s not like, you know, big college parties in the States.

B: It’s not a ranger. It’s just a... It’s like a dinner party. It’s the equivalent of an American dinner party where it’s adults having wine, eating food, okay, leave, you know. But in the States, I think it’s just so common. You rent from a friend, doesn’t matter if the carpet gets dirty or whatever, you know, just, there’s another house over there. There’s plenty of space to go around.

A: Yeah, yeah. That’s a good point. Okay, great.

**Script A.2 (The script of the visual stimuli)**

**Experimenter Effect (Target Video)**

A: One objective of any experiment is, of course, to obtain accurate results. Sometimes, however, problems occur that lead to inaccurate results.

B: What kind of problems?

A: One such problem is the experimenter effect.

B: What exactly does it mean?

A: The experimenter effect occurs when a researcher’s expectations affect the outcome of the experiment.

B: Why does his expectation affect the outcome?

A: The researcher expects a particular result from the experiment, and that expectation causes the researcher to act in ways that influence the behavior of the experiment participants, thus making the results inaccurate.

B: That sounds tricky.

A: For example, I recently read about a case in which a research was given two groups of monkeys and he was asked to train these monkeys to pick up a ball and put it in a box. And he was told to record how many hours it took to train each monkey to learn to do this.

B: Okay, this is an experiment on animal behaviors.

A: Now before he started the training, the researcher was told that one group of monkeys was highly intelligent, and that the other group was less intelligent.

B: But what is the real situation?

A: In truth, there was no difference between them. All the monkey were actually very similar in terms of intelligence.

B: But the researcher didn’t know that.

A: Exactly. He thought one group was smarter. So he expected that that group would be easier to train.

B: So, what happened?

A: Well, the researcher trained the monkeys to perform the action and it turned out that on average it took him two hours less time to train the supposedly smart monkeys than those supposedly less intelligent monkeys.

B: Why did this result occur?

A: Well, it turns out that with the supposedly smart monkeys, the researcher smiled at them a lot, gave them a lot of encouragement, talked to them a lot, worked hard to communicate with them.

B: Positive cues boosted their learning to a large extent. But what about the other group?

A: With the monkeys he thought were less intelligent, he wasn’t as enthusiastic. He didn’t try quite as hard, wasn’t quite as optimistic.

B: No wonder they learned slower.

A: All in all, this study does a good job in demonstrating the existence of the experimenter effect.

# APPENDIX B

**Comprehension Test B.1**

**Things We Miss about America (5 min)**

1. What are the two things that Alicia and Michael miss about America?
A. Oven and convenience store.
B. Turkey and convenience store.
C. Turkey and space.
D. Oven and space.
2. Why does Alicia miss ovens when she’s outside of America?
A. Smaller ovens are more convenient.
B. Ovens in other countries lack variety.
C. American ovens are larger and versatile.
D. Street vendors don't use ovens.
3. How does Michael feel about cooking and ovens?
A. He loves cooking and always uses a big oven.
B. He prefers convenience and doesn’t cook much.
C. He enjoys street food and small ovens.
D. He only goes to bars for parties.
4. Which of the following statements is true?
A. Young people in America usually live with their parents until they get married.
B. Young people in America cannot legally drink alcohol until they are 21.
C. It is more difficult to buy an oven in America than in other countries.
D. You may often be invited to people’s homes when you are in Asian countries.
5. What insight does Alicia provide regarding her social experiences in Asia compared to her experiences in America?
A. Alicia rarely gets invited to house parties in Asia due to cultural differences.
B. Alicia finds that house parties in Asia are more intimate and resemble dinner parties.
C. Alicia’s friends in America are less likely to host house parties than her Asian friends.
D. Social gatherings in Asia are typically larger and more energetic than American house parties.

**Comprehension Test B.2**

**Experimenter Effect (5 min)**

1. What is the experimenter effect?
A. The phenomenon in which experiment participants’ expectations affect the outcome of the experiment.
B. The phenomenon in which the researcher’s expectations affects the outcome of the experiment.
C. The phenomenon in which the test is so easy that most subjects score high.
D. The phenomenon in which the control group reacts beyond the original level in the process of competing with the experimental group.
2. What was the researcher told about these two groups of monkeys before the experiment?
A. One group of monkeys was more intelligent than the other group.
B. One group of monkeys had an obvious IQ deficit.
C. Both groups of monkeys were equally intelligent.
D. The intelligence of monkeys within a group varies greatly.
3. What was the real situation in IQ between the two groups of monkeys?
A. One group of monkeys had an average higher IQ than the other group.
B. One group of monkeys had an obvious IQ deficit.
C. Both group of monkeys had the IQ of a human toddler.
D. There was little difference in IQ between the two groups.
4. What was the difference in the amount of time the researchers spent training the two groups of monkeys?
A. He spent about the same amount of time training two groups of monkeys.
B. He spent one hour less time to train the supposedly smart monkeys than the other group.
C. He spent two hours less time to train the supposedly smart monkeys than the other group.
D. He spent two hours less time to train the supposedly less intelligent monkeys than the other group.
5. In this experiment, why did the outcome become inaccurate?
A. Because one group of monkeys had an undetected IQ deficit.
B. Because the two groups of monkeys were trained too rigorously.
C. Because the two groups of monkeys were trained to pick up a ball in different ways.
D. Because the two groups of monkeys were trained with different effort and optimism.
